# HPV infection and the immune microenvironment in cervical cancer

**DOI:** 10.3389/fimmu.2025.1645019

**Published:** 2025-07-30

**Authors:** Yibo Li, Jingui Deng, Yayong Liu, Shuangfeng Yu

**Affiliations:** ^1^ Department of Scientific Research, Central Hospital Affiliated to Shenyang Medical College, Shenyang, China; ^2^ Department of Obstetrics and Gynecology, Central Hospital Affiliated to Shenyang Medical College, Shenyang, China; ^3^ Department of Microorganism Laboratory, Shenyang Center for Disease Control and Prevention, Shenyang, China

**Keywords:** cervical cancer, immune microenvironment, human papillomavirus, immune pathway, immune checkpoint inhibitors, immunotherapy

## Abstract

Cervical cancer remains a leading cause of cancer-related mortality in women, particularly in low-resource settings, despite advances in treatment modalities. The tumor immune microenvironment (TME) plays a pivotal role in cervical cancer pathogenesis, progression, and therapeutic response, driven largely by persistent HPV infection and subsequent immune evasion mechanisms. Clinical evidence supports the efficacy of pembrolizumab in PD-L1–positive recurrent/metastatic disease, while combinatorial strategies show promise in overcoming resistance. However, challenges persist, including biomarker identification and management of immune-related adverse events. This review elucidates the dynamic interplay between HPV-mediated immune suppression and the TME, highlighting the roles of tumor-associated macrophages (TAMs), regulatory T cells (Tregs), myeloid-derived suppressor cells (MDSCs), and exhausted lymphocyte subsets in fostering an immunosuppressive milieu. Overall, this review integrates current advances in tumor immunology and immunotherapy, providing a comprehensive framework for developing precision-based strategies to improve outcomes in cervical cancer.

## 1 Introduction

Cervical cancer is the fourth deadliest cancer in females globally (1). Stage-dependent management includes hysterectomy or fertility-sparing radical trachelectomy for stage I, often followed by adjuvant radiotherapy and chemotherapy in high-risk cases (2, 3). Stage II requires surgery with tailored adjuvant therapy, while stage III relies on cisplatin-based chemoradiotherapy, occasionally combined with surgery. In stage IVA, treatment is largely palliative, prioritizing symptom control and quality of life. Cisplatin-based chemotherapy continues to play a pivotal role in both symptom management and palliative care (4, 5). Although significant progress has been achieved in the treatment of cervical cancer, the prognosis for patients with locally advanced, recurrent, or metastatic disease remains suboptimal.

Recent advances in tumor immunology have revealed critical mechanisms of immune evasion by cancers, positioning immunotherapy as a transformative approach for cervical cancer, particularly in advanced stages (6). Unlike conventional treatments (surgery, radiotherapy, chemotherapy), immunotherapy enhance endogenous antitumor responses, offering new therapeutic potential. This review summarizes how the cervical cancer immune microenvironment drives pathogenesis, modulates treatment resistance, and influences responses to radiotherapy/chemotherapy, providing a framework for developing novel combinatorial strategies.

## 2 Immune microenvironment in cervical cancer

### 2.1 HPV and the immune microenvironment in cervical cancer

The immune microenvironment of cervical cancer associated with human papillomavirus (HPV) infection exhibits distinctive characteristics (7). Following HPV infection, keratinocytes actively modulate the local milieu, impeding the effective clearance of the virus by immune cells while initiating signal transduction with the host stroma. This interaction promotes a microenvironment conducive to persistent infection, viral dissemination, and cervical cancer progression (8). Evidence demonstrates that TAMs, such as CD68^+^ or CD163^+^ TAMs, show a stepwise increase in expression across cervical tissue, cervical intraepithelial neoplasia (CIN) grades I to III, and invasive cervical cancer. Elevated CD163^+^ TAM counts are correlated with advanced FIGO stages and lymph node metastasis, positioning them as potential biomarkers for cervical cancer progression and dissemination.

Mechanistically, TAMs activated by tumor cells adopt an immunosuppressive phenotype characterized by impaired antigen presentation, suppression of T cell proliferation, and pro-angiogenic activity, all of which facilitate tumor invasion and metastasis (9). Importantly, HPV oncoproteins E6 and E7 directly contribute to the immunosuppressive phenotype of the tumor microenvironment. E6 has been shown to promote PD-L1 expression via activation of the PI3K/Akt signaling pathway, which stabilizes hypoxia-inducible factor 1-alpha (HIF-1α) and enhances PD-L1 transcription (10). Concurrently, E7 activates the STAT3 pathway, further driving PD-L1 upregulation and promoting T cell exhaustion. These mechanisms enable HPV-infected cells to evade immune detection and inhibit cytotoxic T lymphocyte-mediated clearance.

**Figure 1:**
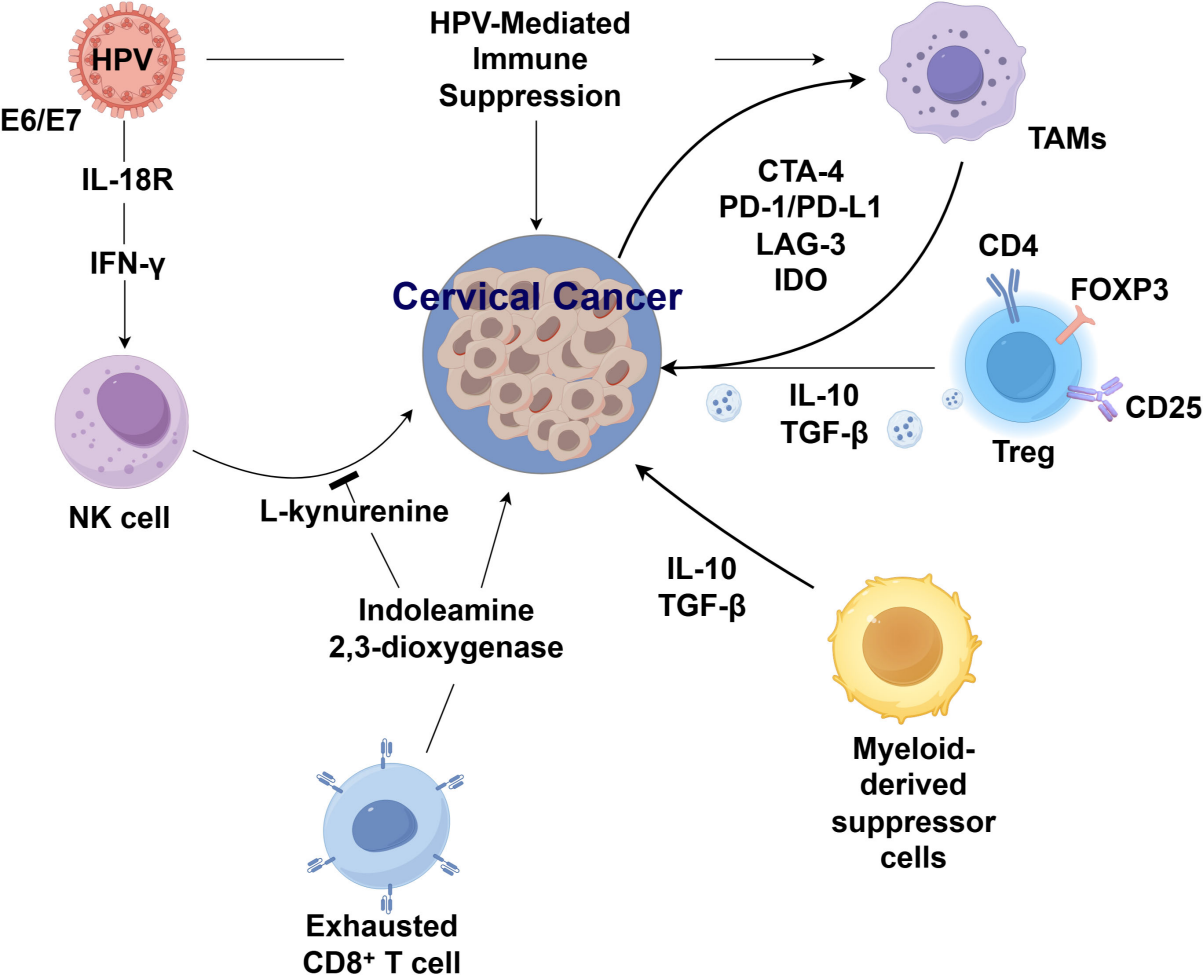
HPV and the immune microenvironment in cervical cancer.

Persistent HPV infection also impairs natural killer (NK) cell function (11). Although CD8^+^ T lymphocyte infiltration is prominent in cervical lesions, these cells fail to suppress malignant proliferation, likely due to HPV-mediated attenuation of immune surveillance and cytotoxic clearance, culminating in an overall immunosuppressive tumor microenvironment (TME) (12). Beyond immune checkpoint regulation, HPV oncoproteins also reprogram metabolic pathways that modulate immune activity (13). Specifically, E7 enhances the expression of indoleamine 2,3-dioxygenase 1 (IDO1) through activation of NF-κB signaling, leading to accelerated tryptophan catabolism and accumulation of the immunosuppressive metabolite kynurenine. Tryptophan depletion inhibits effector T cell proliferation, while kynurenine promotes regulatory T cell expansion, collectively sustaining immune tolerance (14). Interferon-γ (IFN-γ) signaling, which is frequently upregulated in persistent HPV infections, cooperatively interacts with the viral oncoproteins E6/E7 to further upregulate IDO1 expression, thereby exacerbating immunosuppression within the tumor microenvironment. These findings underscore the importance of targeting HPV-induced immune modulation as a therapeutic strategy to improve outcomes, particularly in recurrent and metastatic cervical cancer (**Figure 1**).

### 2.2 Lymphocyte subsets and the cervical cancer immune microenvironment

The immune infiltrate composition in cervical cancer demonstrates significant prognostic and therapeutic implications. Higher CD8^+^ tumor-infiltrating lymphocyte (TIL) density is associated with pelvic lymph node metastasis, whereas recurrent disease shows enrichment of CD8^+^ T cells, CD80^+^CD86^+^ and CD163^+^CD206^+^ macrophages, and FOXP3^+^CD25^+^ Tregs (15). Prognostically, elevated infiltration of CD3^+^, CD4^+^, CD8^+^ T cells along with CD206^+^ macrophages and FOXP3^+^ Tregs correlates with improved progression-free and overall survival in advanced cases (16). In HPV-driven murine models, B cells exhibit dynamic immunomodulatory functions - accumulating in draining lymph nodes while downregulating MHC class II and CD86, yet upregulating PD-L1 and CD39 to suppress T-cell responses and facilitate tumor progression (17). Notably, combined PD-1 blockade and radiotherapy induces clonal expansion of antigen-specific B cells, evidenced by BCR repertoire analysis showing somatic hypermutation and shortened CDR3 regions (18). Single-cell transcriptomics further reveals therapy-induced germinal center B-cell formation, though without concomitant elevation of IgG/IgM responses, suggesting their role as potential biomarkers rather than effector mediators. NK cells also exhibit functionally opposing roles in cervical cancer pathogenesis. On one hand, HPV-encoded E6/E7 bind IL-18R to induce IFN-γ, which is essential for NK cell activation. Conversely, IDO-mediated tryptophan catabolism generates immunosuppressive L-kynurenine that suppresses NK proliferation and cytotoxicity (19).

### 2.3 Other immune cells and the cervical cancer immune microenvironment

TAMs are pivotal immune modulators in the tumor microenvironment (TME) and emerging immunotherapeutic targets in cervical cancer (20). Functionally polarized into M1 (anti-tumor) and M2 (pro-tumor) subsets, TAMs exhibit distinct roles in disease progression (21). While CD68 serves as a general TAM marker, CD163, CD23, and CD204 denote M2 polarization. Clinical studies reveal that both CD68^+^ M1 and CD163^+^ M2 TAMs correlate with lymph node metastasis, with CD163^+^ TAMs further predicting advanced FIGO stage and poor prognosis (22). Myeloid-derived suppressor cells (MDSCs) are another major component of tumor-induced immunosuppression. Both circulating and tumor-infiltrating MDSCs exhibit marked immunosuppressive activity and correlate with cervical cancer stage and metastasis (23). MDSCs comprise granulocytic (G-MDSCs) and monocytic (M-MDSCs) subsets, where elevated G-MDSCs associate with tumor burden and recurrence in early-stage disease, highlighting their biomarker potential (24). Intriguingly, M-MDSCs may synergize with mucosal-associated invariant T (MAIT) cells to facilitate tumor progression (25). Tregs, defined by FOXP3 expression, further shape an immunosuppressive TME. Immunohistochemical analyses of HPV-infected cervical tumors demonstrate upregulated Treg markers (CD25, FOXP3, CD4) and immunosuppressive cytokines (IL-10, TGF-β) in patients with high viral loads or severe infection (26). These findings implicate Tregs in fostering a permissive TME for viral persistence and oncogenesis (26).

## 3 Association between the immune microenvironment and chemoradiotherapy in cervical cancer

Chemoradiotherapy in cervical cancer is intricately linked to the TME, genetic factors, and RNA regulation (27). Radiotherapy, as a primary curative or adjuvant treatment, activates antitumor immunity by triggering DAMP release, inducing type I IFNs, engaging the cGAS–STING axis, and enhancing MHC-I expression. Nonetheless, it concurrently shapes immunosuppressive microenvironment by upregulating PD-L1 and recruiting M2 macrophages and MDSCs (28). To address this duality, several immunomodulatory strategies have been proposed to counteract radiation-induced immunosuppression. For instance, the combination of radiotherapy with immune checkpoint inhibitors such as anti-PD-1/PD-L1 antibodies has shown synergistic effects in reactivating exhausted T cells. Moreover, CSF1R blockade can effectively deplete MDSCs and reprogram tumor-associated macrophages toward a tumor-suppressive (M1-like) phenotype, thereby enhancing antitumor immunity. STING agonists further augment type I interferon responses while reducing myeloid-derived suppressor cell accumulation, representing a promising avenue for combinatorial therapy (10). These strategies are under active investigation in preclinical and early clinical studies, offering actionable paths to overcome TME-mediated radioresistance.

Mori et al. (29) found that radiotherapy enhances CD8^+^ T cell infiltration and PD-L1 expression in tumors, suggesting CD8^+^ T cells as potential biomarkers for radiotherapy response. Disease progression often coincides with immune dysfunction, and radiotherapy-induced myelosuppression may worsen immunosuppression, impacting radio-immunotherapy efficacy. Dynamic monitoring of hematologic toxicity, lymphocyte subsets, and cytokines during treatment can guide immunomodulatory interventions to restore immune function and improve outcomes (30). Radiotherapy may also trigger abscopal effects, where localized irradiation induces regression of distant tumors, likely mediated by immune-driven inflammation and heightened immunogenicity (31). In NACT responders, elevated CD4^+^, CD8^+^, CD20^+^, and CD56^+^ immune markers in the TME suggest chemotherapy-induced immune activation (32). Conversely, Herter et al. (33) observed that chemoradiotherapy reduces T cell counts in tumors and blood while increasing macrophage and neutrophil infiltration. Understanding these immunomodulatory mechanisms is crucial for optimizing combination therapies and enhancing treatment sensitivity in cervical cancer patients.

## 4 Immune microenvironment and immunotherapy in cervical cancer

### 4.1 Fundamental principles of immunotherapy in cervical cancer

Persistent infection with high-risk human papillomavirus (HPV) is the principal etiological factor in cervical carcinogenesis. Although the immune system typically clears HPV-infected cells, malignant transformation enables evasion of immune surveillance, facilitating uncontrolled proliferation and metastatic spread (34). Immunotherapeutic strategies aim to counteract this immune suppression by selectively enhancing antitumor responses. Among these, immune checkpoint inhibitors (ICIs) have emerged as a breakthrough treatment, demonstrating significant clinical potential in cervical cancer (35). ICIs function by disrupting inhibitory signals mediated by immune checkpoint molecules, which tumors co-opt to avoid immune detection. By blocking these pathways, ICIs reinvigorate cytotoxic T-cell activity, restoring their capacity to target and destroy malignant cells (35) (**Supplementary Table S1**). Notably, pembrolizumab, a PD-1-targeting monoclonal antibody, has become the first FDA-approved ICI for advanced, recurrent, or metastatic cervical cancer (36). Its mechanism involves preventing PD-1/PD-L1 interactions, thereby improving outcomes in PD-L1 positive patients and establishing a new therapeutic paradigm (37). Despite this progress, research continues to expand the scope of immunotherapy. Investigations are underway to evaluate other checkpoint targets, such as CTLA-4, and to optimize combination approaches integrating ICIs with conventional therapies (37, 38). As understanding of immune regulation within the TME advances, more precise immunomodulatory strategies are anticipated to further improve treatment efficacy.

### 4.2 Impact of immunotherapy on the immune microenvironment in cervical cancer

Emerging clinical evidence reveals that immunotherapeutic interventions significantly reshape the immune landscape of cervical cancer. In a biomarker analysis of eight patients with advanced/recurrent disease, PD-1 blockade induced robust infiltration of effector lymphocytes (T cells, NK cells, and B cells) within tumor tissue, with enhanced recruitment correlating with treatment response (39). These findings are supported by clinical trial data: the multicenter Phase II C-145–04 study achieved a 44% objective response rate using TIL therapy in heavily pretreated patients, demonstrating durable efficacy at median 3.5-month follow-up (40). Furthermore, a Phase I trial (N=27) established the safety and preliminary efficacy of adoptive TIL transfer following chemoradiation in locally advanced disease (41). Emerging targets like OX40 agonists—which augment CD8^+^/CD4^+^ T cell proliferation and survival—are under investigation (NCT03894618) to broaden therapeutic options (42). T cell exhaustion remains a key resistance mechanism. LAG-3, overexpressed in cervical cancer tissues, suppresses cytotoxic T cell activity and amplifies Treg-mediated immunosuppression (43). Additionally, co-expression of TIM-3 and PD-1 on tumor-infiltrating lymphocytes shows strong association with Treg accumulation and functional impairment of effector cells (44). These insights underscore the potential of combinatorial approaches targeting multiple immune checkpoints to restore T cell competence and improve clinical outcomes.

## 5 Impact of immune pathway regulation on the tumor microenvironment of cervical cancer

The TME represents a complex and finely regulated ecosystem comprising diverse cellular components, signaling molecules, and structural elements (45). This milieu not only provides essential support for tumor growth, invasion, and metastasis but also critically determines the tumor’s response to therapeutic interventions. Cervical cancer is characterized by a particularly immunosuppressive TME, which enables tumor cells to evade immune surveillance and cytotoxic attack (45). The cervical cancer TME encompasses various immunosuppressive cells, notably Tregs and MDSCs, which inhibit T cell activity through the secretion of immunosuppressive cytokines such as transforming growth factor-beta (TGF-β) and IDO (46). In addition, TILs may be rendered dysfunctional or exhausted within this environment, further compromising anti-tumor immune responses (45, 46). Beyond immune cells, stromal components such as fibroblasts and endothelial cells contribute to tumor progression by providing structural support and secreting pro-tumorigenic factors, thereby promoting growth, angiogenesis, and metastatic dissemination (47). These processes facilitate nutrient and oxygen delivery while also generating conduits for cancer cell spread. Immune checkpoint pathways play pivotal roles in shaping this immunosuppressive microenvironment (46, 48, 49). Pathways such as PD-1/PD-L1, CTLA-4, and LAG-3 suppress T cell function and enable immune escape, ultimately accelerating tumor progression and dissemination (48–50).

### 5.1 PD-1/PD-L1 pathway

The foundation of cervical cancer immunotherapy lies in disrupting immune checkpoint pathways that enable HPV-associated tumors to evade host defenses. The PD-1/PD-L1 axis represents a critical immunoregulatory mechanism where PD-1, an inhibitory receptor on T lymphocytes, interacts with its ligand PD-L1 - a transmembrane protein expressed by tumor and immune cells. This interaction transmits immunosuppressive signals that attenuate T cell effector functions, creating an immune-privileged tumor microenvironment (46, 48). In cervical cancer, PD-1/PD-L1 engagement exerts profound inhibitory effects on both CD8^+^ cytotoxic and CD4^+^ helper T cell populations. This suppression manifests through multiple mechanisms: impaired cytotoxic granule release, reduced proliferative capacity, and diminished cytokine production, collectively compromising anti-tumor immunity and facilitating malignant progression (51). The clinical translation of these findings has yielded pembrolizumab, a monoclonal antibody that sterically blocks PD-1/PD-L1 binding. By preventing this immune checkpoint interaction, pembrolizumab restores T cell-mediated tumor recognition and elimination, demonstrating particular efficacy in advanced or recurrent PD-L1-positive cervical cancer cases (46, 48). Current clinical evidence confirms the therapeutic potential of PD-1/PD-L1 blockade, with pembrolizumab emerging as a paradigm-shifting treatment. However, ongoing research seeks to identify complementary biomarkers beyond PD-L1 expression to optimize patient selection and predict treatment response (48, 52). These developments underscore the centrality of PD-1/PD-L1 inhibition in cervical cancer immunotherapy and its capacity to reestablish effective anti-tumor immune surveillance.

### 5.2 CTLA-4 pathway

CTLA-4 represents another critical immune checkpoint pathway gaining attention in cervical cancer therapy. In contrast to PD-1, which primarily functions within peripheral tissues, CTLA-4 predominantly acts in lymphoid organs to suppress early T cell activation and modulate the initiation of immune responses (38, 53). CTLA-4 exerts its immunosuppressive function by competing with CD28 for binding to B7 ligands on antigen-presenting cells, thereby attenuating T cell activation and permitting immune escape (46). Inhibition of CTLA-4 has been shown to restore T cell activity and enhance tumor cell recognition and destruction, thus impeding tumor progression and metastasis (54). CTLA-4 inhibitors, such as ipilimumab, have been developed to block this checkpoint and potentiate anti-tumor immunity (55). Although PD-1 inhibitors are currently first-line agents for cervical cancer immunotherapy, CTLA-4 inhibitors remain under clinical investigation. Ongoing trials are evaluating CTLA-4 blockade either as monotherapy or in combination with other immunotherapeutics, including PD-1 inhibitors (56). Preliminary data suggest that such combinations may yield synergistic anti-tumor effects and improved therapeutic efficacy (55, 57). Despite being in the exploratory phase, CTLA-4 blockade holds significant promise as a future strategy for cervical cancer immunotherapy.

### 5.3 Other immunoregulatory pathways

LAG-3 is another immune checkpoint molecule expressed on T cells, with emerging therapeutic relevance in cervical cancer. Similar to PD-1 and CTLA-4, LAG-3 contributes to T cell suppression within the TME, although through distinct mechanisms (58). Mechanistically, the persistent expression of HPV oncoproteins such as E6 and E7 may upregulate LAG-3 expression via chronic antigenic stimulation, further compounding immune exhaustion. Evidence suggests that elevated LAG-3 expression in tumor-infiltrating lymphocytes may correlate with impaired immune responses in cervical cancer (59). Consequently, LAG-3 blockade represents a potential approach to reinvigorate T cell function and overcome immune suppression (50). While LAG-3-targeted therapies remain in early development and have not yet gained regulatory approval for cervical cancer, preclinical studies have demonstrated promising therapeutic potential (58, 59). Further investigations are needed to elucidate the precise role of LAG-3 in cervical cancer progression and to validate the safety and efficacy of LAG-3 inhibitors in clinical settings. Indoleamine 2,3-dioxygenase (IDO) is an immunosuppressive enzyme that catabolizes tryptophan, an essential amino acid for T cell proliferation and function. Depletion of tryptophan within the TME suppresses T cell activity and facilitates immune evasion. Elevated IDO levels in cervical cancer patients are associated with poor prognosis and contribute to immune suppression by impairing effector T cells and enhancing Treg-mediated tolerance (60). Thus, elucidating the mechanistic role of IDO in cervical cancer may offer critical insights for designing more effective immunotherapeutic strategies.

Transforming growth factor-beta (TGF-β) is a multifunctional cytokine with paradoxical roles in cervical cancer. During early carcinogenesis, TGF-β exhibits tumor-suppressive properties by inhibiting cell proliferation and promoting differentiation. However, as the disease progresses, TGF-β signaling transitions toward a pro-tumorigenic role, enhancing cell motility and invasiveness, thereby facilitating metastasis (61). TGF-β is also implicated in the expansion and activation of Tregs, contributing to a tolerogenic immune milieu. Increased TGF-β levels in HPV-infected cervical tissues promote FOXP3^+^ Treg proliferation, which suppresses effector T cell responses and facilitates immune escape. Thus, HPV-associated TGF-β signaling not only fosters epithelial transformation but also promotes an immunosuppressive microenvironment that limits the efficacy of antitumor immunity and immunotherapy. Moreover, TGF-β modulates the TME by suppressing immune cell activation and promoting immune escape, further exacerbating tumor progression. At advanced stages, TGF-β promotes metastasis through multiple mechanisms. Deciphering the context-dependent roles of TGF-β across cancer stages and developing strategies to selectively target its pro-tumorigenic functions—while preserving its tumor-suppressive activity—may offer novel therapeutic opportunities for cervical cancer.

## 6 Conclusion

Cervical cancer, driven by persistent HPV infection, exemplifies the critical role of the tumor immune microenvironment (TME) in disease progression and therapeutic resistance. The immunosuppressive TME, shaped by HPV oncoproteins E6 and E7, is characterized by infiltrating TAMs, Tregs, MDSCs, and exhausted lymphocytes, which collectively foster immune evasion and limit treatment efficacy. Immune checkpoint pathways, particularly PD-1/PD-L1, have emerged as pivotal therapeutic targets, with pembrolizumab demonstrating clinical benefit in advanced disease. However, response heterogeneity highlights the need for biomarker-driven strategies and combinatorial approaches, such as integrating ICIs with radiotherapy or adoptive cell therapy, to overcome resistance and enhance antitumor immunity.

Advancing cervical cancer immunotherapy requires deeper mechanistic insights into TME dynamics, including spatial remodeling post-therapy and the role of novel targets like OX40 and TIM-3. Clinical trials should prioritize adaptive designs to optimize sequencing and safety of multimodal regimens, while addressing disparities in resource-limited settings. By leveraging the TME as both a therapeutic target and a modulator of response, these efforts hold promise for improving outcomes, particularly in recurrent or metastatic disease, and reducing the global burden of cervical cancer.
